# The short-term effects of the implementation of the "Treat All" guidelines on ART service delivery costs in Namibia

**DOI:** 10.1371/journal.pone.0228135

**Published:** 2020-01-27

**Authors:** Carl Schutte, Steven Forsythe, Johnface Fedes Mdala, Brady Zieman, Rachael Linder, Lung Vu

**Affiliations:** 1 Genesis Analytics, Johannesburg, South Africa; 2 Avenir Health, Glastonbury, Fountain Hills, AZ, United States of America; 3 IntraHealth, Windhoek, Namibia; 4 Population Council, Washington, DC, United States of America; London School of Hygiene and Tropical Medicine, UNITED KINGDOM

## Abstract

The introduction of “Treat All” (TA) has been promoted to increase the effectiveness of HIV/AIDS treatment by having patients initiate antiretroviral therapy at an earlier stage of their illness. The impact of introducing TA on the unit cost of treatment has been less clear. The following study evaluated how costs changed after Namibia’s introduction of TA in April 2017. A two-year analysis assessed the costs of antiretroviral therapy (ART) during the 12 months before TA (Phase I–April 1, 2016 to March 31, 2017) and the 12 months following (Phase II–April 1, 2017 to March 31, 2018). The analysis involved interviewing staff at ten facilities throughout Namibia, collecting data on resources utilized in the treatment of ART patients and analyzing how costs changed before and after the introduction of TA. An analysis of treatment costs indicated that the unit cost of treatment declined from USD360 per patient per year in Phase I to USD301 per patient per year in Phase II, a reduction of 16%. This decline in unit costs was driven by 3 factors: 1) shifts in antiretroviral (ARV) regimens that resulted in lower costs for drugs and consumables, 2) negotiated reductions in the cost of viral load tests and 3) declines in personnel costs. It is unlikely that the first two of these factors were significantly influenced by the introduction of TA. It is unclear if TA might have had an influence on personnel costs. The reduction in personnel costs may have either represented a positive development (fewer personnel costs associated with increased numbers of healthier patients and fewer visits required) or alternatively may reflect constraints in Namibia’s staffing. Prior to this study, it was expected that the introduction of TA would lead to a significant increase in the number of ART patients. However, there was less than a 4% increase in the number of adult patients at the 10 studied facilities. From a financial point of view, TA did not significantly increase the resources required in the ten sampled facilities, either by raising unit costs or significantly increasing the number of ART patients.

## Introduction

### Evolution of ART guidelines

In 2003, the World Health Organization (WHO) produced a set of treatment guidelines for resource limited settings, as much of the global epidemic was concentrated in sub-Saharan Africa [1]. These guidelines recommended a cluster of differentiation 4 (CD4) threshold of 200 cells/μL for most patients, specified first line and second line anti-retroviral therapy (ART) regimens and outlined approaches for simplifying clinical care for HIV-positive patients.

Throughout the early 2000s, several observational studies were published that demonstrated individuals on ART were less infectious than those not on treatment [2–5]. Researchers found that, among sero-discordant couples, HIV-positive partners who began treatment immediately had a far lower HIV transmission rate than those who waited until a CD4 count of 250 cells/μL had been reached [6]. Two randomized control trials [7,8] demonstrated that earlier ART initiation resulted in reduced mortality and morbidity. In response, the WHO guidelines were updated in 2010 to recommend ART initiation for those with a CD4 count of 350 cells/μL or lower [9] and in 2012, WHO recommended immediate ART for HIV-positive people with HIV-negative partners to prevent transmission [10].

By 2013, the WHO issued consolidated guidance on ART and recommended that ART should be given to people living with HIV who had a CD4 count of less than 500 cells/μL [11].

In 2014, the Joint United Nations Programme on HIV/AIDS (UNAIDS) introduced the 90-90-90 targets as part of their Fast-Track global strategy, providing a benchmark to guide countries as they work towards HIV epidemic control [12]. In 2015, WHO recommended immediate initiation on treatment, or what is now referred to as “Treat All” (TA) [13] (also referred to as “Test and Start” and “Test and Treat”). This approach to initiating treatment was based on findings by several observational studies indicating that universal ART showed significant increases in ART uptake, linkage to care and increases in median CD4 values [14–16]. The 2015 WHO guidelines also noted that rapid implementation of the recommendations would aid countries in achieving the ambitious 90–90–90 targets by 2020 and the end of the AIDS epidemic by 2030. As of July 2018, 84% of low and middle-income countries (LMIC) and 100% of Fast-Track countries had adopted a TA policy, while another 5% of all LMIC planned to adopt TA before the end of 2018.

### Economic rationale for TA

Achieving TA may require significantly increasing investments in treatment, especially in high prevalence countries. In a low prevalence country such as Rwanda (2.5% prevalence), the gap in achieving the targeted level of viral suppression is only 1,800 patients. However, in South Africa, where HIV prevalence is 20.4%, there are 1 million people who still need to be virally suppressed to achieve the final 90 target.[17]

In a review of 12 models using various CD4 thresholds for ART initiation, researchers noted that investing in earlier ART eligibility should be regarded as a long-term investment in overall population health. Though upfront costs can be high, the health benefits over time, such as the cost of averting poor health and premature death, become progressively lower as costs and benefits are looked at over longer time periods. Among models from South Africa, Zambia, India and Vietnam, authors found that earlier ART eligibility was very cost effective in low- and middle-income countries [18]. The cost per disability adjusted life year (DALY) averted for changing eligibility to all HIV-positive adults from a CD4 of 350 cells/μL or less, ranged from USD438 to USD3,790, and in Vietnam and India, expanding ART to all HIV-positive adults resulted in an incremental cost-effectiveness ratio (ICER) of USD289 and USD131 per DALY averted, respectively.

Multiple studies have been conducted in, or modeled after, the epidemic in South Africa. One study found that TA among adults was found to be more cost-effective, at USD160-USD220/quality adjusted life years (QALY), compared to ART for CD4 cell counts ≤350 cells/μL, or to pre-exposure prophylaxis (PrEP) for the general population [19]. Another study looked at expedited ART initiation among pregnant women and found it to be very cost-effective compared to standard services (USD1,160 per QALY saved) [20]. A study using point-of-care tests found that same day initiation was more effective and only 4% more expensive (a difference of USD18 per virally suppressed patient) than standard initiation for patients achieving viral suppression [21].

An additional model based on the HPTN052 trial found that TA among serodiscordant couples in South Africa and India was cost-effective over a lifetime; USD590 per life-year saved, and USD530 per life-year saved, respectively [22]. Finally, a study from China demonstrated that the unit cost for an additional patient receiving immediate ART was USD83.80, declining to USD9.69 in the second year; which represented an effective and sustainable intervention, according to the study investigators [23].

The cost of TA, as one component of differentiated service delivery models, has also been assessed [24]. Costs of a streamlined care model were evaluated among 17 clinics in Uganda and Kenya, in which immediate ART initiation was one component of an overall model. The estimated annual per patient costs receiving the complete model of care were USD291 per person per year. On a larger scale, it is estimated that from 2015–2050, in nine sub-Saharan African countries with the highest HIV burden, USD261 billion will be needed for universal TA, including costs for scale-up of prevention services to account for population growth [25].

### HIV in Namibia

Namibia has a population of approximately 2.4 million people and is characterized by a sparsely populated landscape and widespread socio-economic inequities [26]. A highly mobile population, often living in underdeveloped, hard to reach communities, tends to increase the risk of vulnerability to diseases such as HIV and AIDS. Although 13% of government expenditures are dedicated to healthcare [27], high HIV prevalence and incidence remains. Namibia has one of the world’s highest HIV prevalence rates, with an estimated 11.8% of adults (15–49) living with the disease [28]. Approximately 200,000 adults and children are HIV-positive in Namibia today. Namibia’s prevention and treatment programs have reduced HIV incidence rates by 50% since 2012, dropping to 0.36% in 2017 [29]. The epidemic is generalized in terms of transmission, and prevalence tends to be higher among women compared to men of the same age group [30].

Regional variability of HIV prevalence fluctuates widely across Namibia; ranging from 7% in the Omaheke region to 24% in the Zambezi region, with most people living with HIV (PLHIV) in rural, hard to reach areas [31]. Due to this geographic distribution, and the need to rapidly and broadly scale-up testing and treatment, the Government of Namibia is transitioning towards increased community-based care and support for PLHIV and shifting much of the management and responsibility of ART from physicians to nurses.

The Government of Namibia has made significant strides in terms of HIV treatment and care. In 2016, the Government revised national treatment guidelines to align with the WHO recommendation of beginning ART immediately after a positive HIV test result [32]. The TA guidelines were officially implemented on 1 April 2017. To help reach as many patients as possible and to improve adherence, nurses were trained to provide ART (rather than being delegated to only physicians) and are supported by a team of community health workers linking services between health facilities and the community.

**Fig 1:**
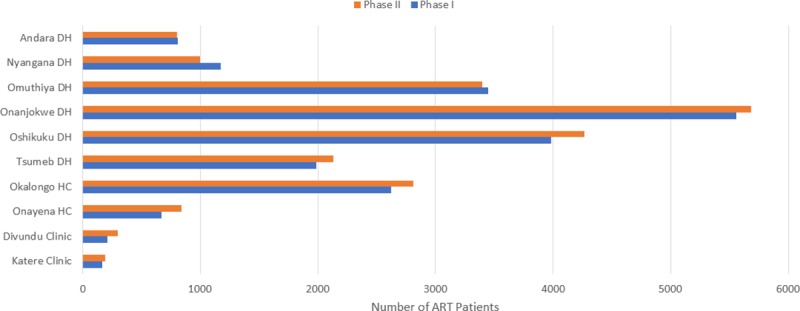
Namibia’s cascade.

Among the 200,000 people living with HIV in Namibia in 2018, 180,000 knew their status (90%), almost all of whom are receiving ART [30]. Data from 2018 also indicate that 170,000 Namibians have achieved viral suppression, or 87% of all people living with HIV [33]. Namibia is the first country to have three quarters of its HIV-positive population virally suppressed [34], a strong indicator of a successful HIV treatment program. Fig 1 illustrates the significant achievement that Namibia has made in terms of testing, treatment and viral suppression, exceeding the target of having 73% of all HIV positive persons virally suppressed by 2020.

In 2017, Namibia spent nearly USD283 million on the country’s HIV/AIDS response [28]. Forty-four percent of the resources for Namibia’s HIV/AIDS response are derived from domestic public sources (USD124.5 million). This is followed by international sources, which account for 29% of all HIV/AIDS resources (USD83.4 million). Domestic private resources account for the remaining 26% of all funds.

However, significant barriers remain. In 2016, only 54% of health facilities providing ART services reported an adherence rate, among patients, of greater than 75%. Additionally, retention rates decreased from 80.2% in 2014, to 75.5% in 2016, and there were an estimated 2,700 AIDS-related deaths among adults and children in 2017. In addition, approximately a quarter of all HIV-positive Namibians are currently not virally suppressed, which contributes to worse health outcomes and increased new infections.

### Study rationale and purpose

Namibia started implementing the TA guidelines nationwide on incorporated TA ART policies in April 2017 [32]. There is a critical need in understanding how the TA implementation may affect the patient and health systems outcomes as well as the country’s progress toward the 90-90-90 targets. In response, Project SOAR and USAID-Namibia, in partnership with the Government of Namibia and IntraHealth International, conducted a mixed method implementation science research study to better understand the impact of TA on ART initiation and viral suppression.

The analyses also assessed the short-term financial impact of the change to TA by estimating the annual cost per ART-patient before and after the implementation of TA policies, at both the district hospital, health center and clinic levels. Results from this costing study contribute to the small but growing body of research and evidence of the impact of TA over time, while helping to inform efficient resource planning and allocation as the Government of Namibia scales up and expands HIV services.

## Materials and methods

### Human subjects

The study was approved by Population Council Review Board (New York, USA) and the Ministry of Health and Social Services (Namibia). All data collectors were trained in research ethics and signed confidentiality agreements. No personal identifying information was obtained during data collection, including names associated with patient records.

### Costing approach

The costing component of the study calculates the *unit* cost, described in more detail below, of providing ART services including costs for the 12 months before the adoption of TA (April 2016-March 2017) and the 12 months after the adoption of TA (April 2017-March 2018) at 10 health facilities in northern Namibia.

#### Perspective

The cost analysis took a health sector provider perspective. Patient costs, such as transportation and lost opportunity costs associated with visits to the facility, were not included as these were not the focus of this analysis. The perspective focused on the average cost of treatment, not the incremental cost of TA.

#### Financial vs. economic costing

“Financial costs capture the resources that are ‘paid’ for. They are thus contingent on the extent to which payment is made for the resources used. In cases where resources are donated, they would not be included in financial costs. Thus, financial costs can be generalized only across settings with similar payment structures. Also, since all resources (even donated) are paid by someone, financial costing implies a specific payer perspective–i.e., the financial cost from the point of view of an identified person, program, or organization.”[35] Conversely, economic costing reflects the true economic or opportunity costs of an intervention. For the purposes of this costing, financial costing was used to calculate the unit costs and includes equipment costs depreciated equally over the useful life of equipment and assets.

#### Data collection process

Data collection activities were carried out by 2 trained data collectors with supervision from an experienced costing consultant. The costing data collection comprised interviewing numerous key informants including service delivery staff at facilities, administrative support staff, stores and pharmacy staff and district team members. At the same time, implementing partner program staff assisted by compiling output data from the electronic ART patient monitoring system and general hospital records, securing pharmaceutical and health commodity issue records from central medical stores and central procurement at the Ministry of Health and Social Services (MOHSS).

#### Sampling

Study sites were ten facilities that received technical assistance for HIV clinical services from IntraHealth International under the USAID Technical Assistance Project (UTAP) since 2015 and were purposively selected. The selection of the sites was carefully consulted with USAID and local stakeholders, aiming to capture large and medium size facilities.

Detailed information of the ten selected sites are shown in Table 1. These sites comprise six district hospitals, two health centers and two clinics. The number of ART patients varies significantly between the ten sites ranging from an average of 5,894 patients at Onadjokwe District Hospital to an average of only 206 patients at Katere clinic for the Phase II period.

**Table 1:** Summary of selected facilities and number of adult and pediatric ART patients.

Facility	Region	Urbanization	# Phase I Patients	# Phase II Patients
Okalongo Health Centre	Omusati	Peri-Urban	3,090	2,814
Oshikuku District Hospital	Omusati	Urban	4,359	4,270
Omuthiya District Hospital	Oshikoto	Urban	3,824	3,615
Onayena Health Centre	Oshikoto	Village	790	917
Onandjokwe District Hospital	Oshikoto	Rural	6,039	5,894
Tsumeb District Hospital	Oshikoto	Urban	2,078	2,238
Divundu Clinic	Kavango East	Peri-Urban	217	314
Andara District Hospital	Kavango East	Peri-Urban	933	869
Nyangana District Hospital	Kavango East	Rural	1,267	1,070
Katere Clinic	Kavango East	Village	180	206

#### Units

The “units” in the unit cost analysis are the average number of patients on ART (adult and pediatric) in each Phase. Although these data were collected at the facility level, data obtained from the electronic patient management system (EPMS) were deemed to be more accurate and reliable. Patient numbers were shared and discussed with IntraHealth (which provided technical assistance, including monitoring and evaluation data capturing and reporting) program staff as a means of validation. In addition to the ART patient numbers, the EPMS was also used to arrive at the total number of ART visits for each facility. ART visits, as a proportion of total outpatient visits, were used to allocate maintenance and utility costs between ART and other services. Total outpatient visits and in-patient days were obtained from the routine monitoring and evaluation systems at the facilities.

#### Retrospective vs. prospective costing

Retrospective costing is an approach for assessing historical costs and cost changes, while prospective costing monitors costs as the services are being delivered [36]. A retrospective costing approach was used to calculate the costs. This approach included a review of government expenditure records where these were available and sufficiently detailed. The primary unit of measure comprised an annual unit cost per patient receiving ART-related services excluding HIV testing and counselling. In other words, the annual unit cost per patient receiving ART-related services amounted to the total cost of providing treatment divided by the average number of patients treated.

#### Scope and method for allocating resource use

The costing was required to evaluate costs in pre-defined cost categories. These cost categories are described below.

*Consumable supplies costs*. The most important health commodities included anti-retroviral drugs (ARVs), drugs to prevent and treat opportunistic infections (OI), and non-pharmaceutical health commodities such as gloves and masks. Due to the absence of accurate stock records at individual sites, and therefore accurate consumption records by facilities, the cost of ARVs was determined by using the mix of ARV regimens for each facility derived from the electronic dispensing tool (EDT). The average prices derived from central medical stores (CMS) stock issue records were then applied to the regime mix referred to above and the average number of ART patients for Phase I and II. CMS issue prices represent the charge to the facilities. The cost estimates for OI drugs and other health commodities were based on quantities provided by facility staff and prices from CMS stock issue records. The list of OI drugs include Isoniazid for prophylaxis but did not include the cost of treating TB in ART patients.

*Direct personnel costs*. For the purposes of determining human resource costs, the salaries paid to staff were based on published government scales and validated during the interviews [37]. Based on an analysis of the salaries and benefits received by most staff, a further 33% was added to basic published salary scales to provide for social services including medical aid and other travel and housing benefits. The 33% was based on an analysis of actual fringe benefits due to government health staff based both in interview data and government directive. These vary between different cadre and 33% was considered a representative average for the dominant cadre in the health facilities.

In order to quantify the cost of personnel attributable to HIV/AIDS treatment, the data collectors asked the respondents to determine the percentage of time that each staff member spent on the ART program. In many cases staff had been deployed permanently to the ART clinic and all their costs were included in the ART unit costs. Where this was not the case only the relevant portion of staff time was included in the analysis.

*Training costs*. All ART-related training was included in the costing. An ingredients-based approach was used to estimate the cost of training for resident-based training and training events where no accommodation was provided. Training costs included accommodation, cost of facilitation, materials, per diem and travel allowances. Costs were provided by IntraHealth, which facilitated many of the training events.

*Transport costs*. The most significant ART-related travel and transport cost comprised travelling by district and clinic teams to either provide outreach services at outreach points and to visit clinics to provide support and oversight and for monitoring and evaluation purposes. It was not possible to accurately calculate the share of travel and transport costs to be allocated to each specific facility because logbooks recorded trips but typically not the purpose of the trip. Many trips combined visits to several destinations and outreach trips could be for multiple purposes (ART, HIV testing and counselling, tuberculosis treatment and male circumcision). A further complication is that there appeared to be some inconsistency about where the ART patients benefiting from the outreach visits were registered and it was not possible to split the patient data between those receiving services at outreach points and those at health facilities. Where outreach points are not clinics and patients are included in the district hospital register, some cost should be absorbed by the ART patients at that hospital.

In order to calculate some travel costs, a certain number of weekly trips were assumed to each clinic and health center and these costs were included in the ART unit costs of those facilities receiving support. This approach and the principle that the beneficiary facility should absorb the cost was agreed with the steering committee.

*Laboratory costs*. Laboratory costs were based on the reported number of tests from each facility (tests costed include CD4, viral load tests, creatinine, blood chemistry (ALT) and hemoglobin). The total number of tests was calculated by extrapolating the sample to the total population of ART patients at each facility and applying the published tests prices available from the National Institute of Pathology (NIP) website. It is acknowledged that these represent an acceptable proxy for the cost to the MOHSS. In the absence of access to detailed NIP laboratory price and consumption records, it was not possible to tell exactly when the price decreases were reflected in billing records. What is known is that the MOHSS was engaged in ongoing negotiations with the NIP to reduce the cost of viral load tests. The first price decrease occurred in August 2017. It is probable that the *average* price for the Phase II year of viral load tests was higher than the cost used in the study. It is the researchers view given the considerable and permanent reduction in the cost of viral load tests, that use of the current price for calculating unit costs renders the results more useful for planning purposes.

*Capital costs*. Data collectors listed all equipment items used by the ART program during their facility visits. Where rooms were shared, the assets in those rooms were shared based on the time rooms were used for ART. The value of equipment and useful life data were derived from a list of equipment costs provided by central procurement at MOHSS. Capital costs were annualized over the estimated useful life of equipment items without discounting. In a few cases where costs were not provided, estimates of costs were obtained from local vendors and the useful life for most items were based on estimates provided by the MOHSS, and in some cases on WHO Choice or American Hospital Association guidelines.

All buildings and rooms used for ART were measured and included in the costing by applying an ‘all-in’ commercial rental obtained from local estate agents active in the commercial market. This rental was deemed to be a sound proxy for the cost of owning and maintaining buildings in these remote locations. Where rooms were shared, the cost of buildings was split based on a time allocation provided by facility staff.

*Utility and maintenance costs*. Utility and maintenance costs in this study comprise the operational costs associated with operating the health facilities and creating a functional environment for delivery services. In some cases, these include outsourced services. As with many government accounting systems, these costs are pooled at district level and are not allocated to specific facilities or health programs. Utility costs comprising mainly electricity, water, communication and security costs were extracted from source documents as a means of isolating facility costs and were then allocated to the ART program based on patient visits.

*Support personnel costs*. Several support personnel provide support to the ART program. These included reception staff, cleaners, data capture and administrative staff who spend time on the ART program. Costs were allocated based on their share of time spent on the ART program and, where it was not possible to determine a time-spent allocation, general support staff costs were allocated based on the total number of ART visits as a proportion of total facility outpatient equivalent visits. As noted above, published salary scales were used to calculate personnel costs [37].

*Management and supervision costs*. Each district is also supported by a district management team which provides high-level support services to all the facilities in the districts. These teams typically include the Senior Medical Officer, Nurse Mentors, Control Officers, human resource and administrative support, Monitoring and Evaluation Officers and data-capture clerks. The cost of these district teams was allocated first to the facility, based on facility outpatients when compared to total district outpatients and to the ART program using the number of ART visits as the allocation factor. As with other human resources, the published salary scale was used to calculate the cost of management and supervision [37].

#### Inflation and conversion rates

For equipment and some non-ARV pharmaceuticals, a price list was not provided during the Phase II data collection period. These costs were inflated by 6.15% from Phase I to utilize in Phase II. The inflation rate used was the average inflation rate in Namibia for 2017 (https://www.statista.com/statistics/510131/inflation-rate-in-namibia/). For all other items, the actual price of the item in Phase I and Phase II were obtained.

The currency exchange rate used for the conversion of local currency units to USD were derived by calculating the average rate applicable during Phase I. An exchange rate of N$14.12 to the US dollar was therefore used (www.oanda.com/currency/converter). While this exchange rate was used throughout the study, the exchange rate during Phase II changed to N$12.60 to the US dollar. However, the Namibian dollar has subsequently weakened since the conclusion of Phase II, suggesting that the use of the Phase I exchange rate is probably consistent with current rates.

#### Data collection, cleaning and entry

Data collection, cleaning and capture comprised: 1) the organization and cleaning of data received from the data collection (data included cost data, including quantity used and input costs, from each of the ten facilities, as well as data on the number of ART patients), 2) the examination of the consistency of this data with that derived from the survey sample and national-level data and 3) populating an excel spreadsheet with the relevant data. Data was collected using a detailed, structured hard-copy data collection tool. This tool facilitated the collection of facility contextual and output data and data for all cost elements; these included clinical and non-clinical personnel numbers, pay grades and time spent on ART, number of laboratory tests, training, transport, equipment, building, health commodities consumed, overhead and external services costs. After an initial review and cleaning, all the responses in the hardcopy questionnaires were captured into an electronic version of the hard-copy questionnaire. A separate costing Excel workbook was completed for each health facility for Phase I and II using data from the questionnaire and from other sources such as the government salary scales which were obtained centrally. Once these tasks had been completed, an Excel worksheet was compiled with all the unit costs for each facility for both phases to facilitate the comparative analysis.

The analysis of cost data focused on examining the distribution of costs by cost category, comparing the unit costs from Phase I with II, further analyzing significant differences to understand the underlying drivers for changes in cost and exploring certain sensitivities.

## Results and discussion

### Number of adult patients and visits from Phase I to Phase II

The number of adult ART patients for Phase I year in the 10 sampled facilities was 20,626. During Phase II, adult patients increased to 21,408. The increase in adult patient numbers is expected given the implementation of TA on 1 April 2017. Discussions with facility and IntraHealth program staff during data collection suggested that some facilities implemented the guidelines before the official launch of 1 April 2017. This would explain why the increase in adult patient numbers during Phase II is not more significant as many newly eligible patients would have been initiated on treatment before the change-over. In addition to introducing the new treatment guidelines, an ongoing process of decentralization has resulted in the transfer of patients to lower-level health facilities which may have offset any increase in the number of patients at certain facilities.

**Fig 2:**
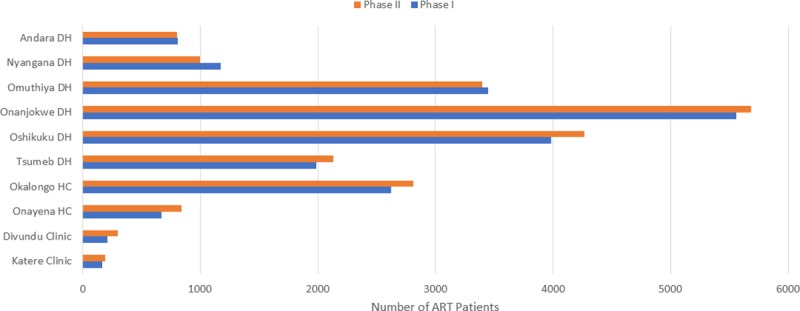
Overview of adult patient numbers at sampled facilities for Phase I and II.

Fig 2 shows the number of adult patients enrolled at the sampled facilities, averaged over the 12 months for Phase I and Phase II. Most facilities except Nyangana and Omuthiya reflect modest increases in patient numbers. The figure also highlights the significant difference in patient loads between large district hospitals (Onandjokwe had 5,681 adult patients in Phase II) and the small, remote clinics of Katere and Divundu where patient numbers were 191 and 296 adult patients respectively for Phase II. Okalongo Health Center also has more ART patients than Tsumeb, Nyangana and Andara district hospitals.

The total number of estimated visits by ART patients was used during the costing to allocate some shared costs; mainly maintenance and utility and support personnel costs. In other words, the proportion of total facility ART patient visits expressed as a percentage of total facility or district outpatient visits, was used to calculate the facilities share of some maintenance and utility and support personnel costs.

### Summary of unit costs in Phases I and II

The unit cost per ART patient, weighted by volume and using a constant exchange rate (N$14.12 = USD1) for Phase I and Phase II were USD360 (N$5,078) and USD301 (N$4,245) respectively (Table 2). The unit cost decreased by 16% between Phase I and Phase II.

**Table 2:** Summary of unit costs.

Description	Phase I	Phase II
Unit cost per patient (weighted by volume, N$)	N$5,078	N$4,245
Unit cost per patient (weighted by volume USD, using constant exchange rate)	$360	$301
Total number of adult patients	20,626	21,408

**Fig 3:**
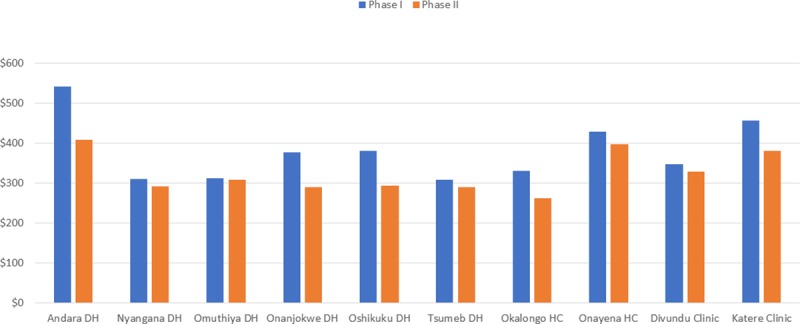
Unit costs for all health facilities.

An analysis of the average unit cost by facility indicates a reduction in unit costs for all facilities when comparing Phase I with Phase II. The reduction in unit costs is, however, most pronounced in five facilities: 1) Andara District Hospital, 2) Onandjokwe District Hospital, 3) Oshikuku District Hospital, 4) Okalongo Health Center and 5) Katere Clinic. The reduction in unit cost in all five facilities was due to: 1) reduced ARV prices, 2) lower negotiated costs for viral load tests, and 3) a reduction in human resource costs. In the other health facilities, the decreases in unit costs were modest. The unit cost for each facility is reflected in Fig 3.

### Unit cost by component

#### Analysis of cost components as a share of the average unit cost

An analysis of the unit costs by component (Table 3) highlights that for both Phase I and Phase II, ARVs and other consumables remain the biggest cost component of treatment costs at 37.8% and 40.2%, respectively. The share of total costs attributable to personnel and laboratory costs are also very similar when comparing the unit costs from Phase I and Phase II. Personnel comprise 21.6% and 22.3% of total costs for Phase I and Phase II, respectively, while laboratory costs were 31.9% and 28.9% for Phase I and Phase II.

**Table 3:** Unit costs by cost component.

	Phase I		Phase II	
	Cost (US$)	%	Cost (US$)	%
Consumable Supplies Costs	$135.00	37.8%	$ 120.89	40.2%
Direct Personnel Costs	$77.77	21.6%	$ 67.07	22.3%
Training Costs	$1.82	0.5%	$ 0.91	0.3%
Transport	$1.28	0.4%	$ 1.31	0.4%
Laboratory Costs	$114.82	31.9%	$ 86.81	28.9%
Capital Costs	$0.77	0.2%	$ 0.64	0.2%
Maintenance/Utility Costs	$12.60	3.5%	$ 7.62	2.5%
Support Personnel Costs	$11.40	3.2%	$ 12.08	4.0%
Managements Supervision Costs	$3.16	0.9%	$ 3.29	1.1%
Total	$359.61	100.0%	$300.62	100.0%

#### Analysis of the change in value of cost components

This section present changes in cost per category from Phase I with Phase II. As noted in Table 3, the weighted average reduction in unit cost between the two phases was USD59 (if the actual Phase II exchange rate had been used in Phase II, this difference would have been reduced to only USD12). Three components account for this reduction in the unit costs; these are consumable supplies including ARVs, personnel costs, and laboratory costs. Although many of the other smaller costs also decreased or increased in the case of support personnel and management and supervision costs, the magnitude of these changes is insignificant and had little impact on the change in the unit cost.

*Consumable supplies including ARVs*. Consumable supplies decreased by USD15 when comparing Phase I with Phase II. A closer examination of the change in the cost of ARVs indicates that there was a small increase in the cost of ARVs due to a shift from TDF/3TC/EFV first line regimen to TDF/FTC/EFV. In the sampled facilities the proportion of adult patients on the latter regimen increased from 65% during Phase I to 78% during Phase II. As this is a slightly more costly regimen, the unit cost was estimated to increase slightly because of this change. However, the decrease in the cost of the five most frequently used adult regimens, which cover over 90% of regimens used, is estimated to have resulted in a decline in the unit cost of over USD21 and negated the increase referred to above. As an example, the annual costs of the TDF/FTC/EFV regimen, declined from USD119 to USD92 using a constant exchange rate.

*Laboratory costs*. The reduction in laboratory costs accounted for USD28 of the reductions in cost and is the net result of an increase in laboratory testing and a significant decrease in the cost of each viral load test. Results from exit interviews extrapolated to the facility population indicated that the number of laboratory tests all increased except for the creatinine test. Viral load tests increased from 0.51 tests per patient during Phase I to 0.65 tests per patient during Phase II. CD4 count tests also reflected an increase during Phase II. With the limited information available, it is estimated that the increase in the number of tests using Phase I test prices (the quantity variance) accounted for an increase of approximately USD28 per patient. If laboratory test prices had stayed the same as in Phase I, the increase in the *number* of laboratory tests would have resulted in an increase of USD28 in the unit cost. However, the cost of laboratory tests, notably the cost of viral load tests, reduced significantly and more than offset any increase that resulted from increased tests. The increase is offset by the substantial decrease in the unit cost of the viral load tests of USD73, the effect of which is not only to absorb the increase but to generate the substantial decrease in the unit costs of laboratory tests. It is likely that the actual *average* price for viral load tests for the Phase II year was higher than the cost used in the study and that the reduction in the unit costs of laboratory tests was less significant than depicted. However, the reduction in the cost of viral load tests is currently in place after having been successfully negotiated by the MOHSS. Therefore, the cost savings are probably indicative of future costs.

*Personnel costs*. The reduction in personnel costs comprised a USD11 decrease in the unit cost between Phase I and II. The cost of personnel is affected by the number of staff deployed to support ART services, the amount of time allocated by staff to the ART program, remuneration levels and by the mix of staff. In order to examine if the amount of time allocated to ART services had reduced on average the time allocations for all staff were converted to full time equivalents (FTE). This calculation highlighted that there was a significant reduction in the number of staff time allocated to ART services, which was primarily responsible for the decline in the personnel unit costs. On average FTE staff per facility reduced from 14.3 to 10.5 FTE per facility. The reduction in FTE was concentrated in four facilities: Andara, Okalongo, Oshikuku, and Tsumeb facilities. The reason for these reductions is not well understood but could relate to a combination of increased decentralization and community-based support, more efficient use of staff, vacancies and reduced ART patient visits (observed from exit interviews in the clinical study component). Given an annual increment of 9% for government salaries, the increase in salary cost of 6% per FTE in the costing study, points to a shift in staff time from senior to more junior staff.

## Conclusions

The estimated cost of treatment for Phase II (USD301 per patient per year) is generally consistent with other recent studies on treatment costs in neighboring countries. For example, data from South Africa indicated that the cost of treatment was USD300 per patient per year [38]. A multi-country study by the Clinton Health Access Initiate estimated annual treatment costs of USD232 per year in Rwanda, USD136 in Malawi, USD186 in Malawi, USD278 in Zambia, and USD682 in South Africa [39].

### Changes in treatment costs with the introduction of TA

The study found that the transition from the existing standard of care to TA corresponded with a small decrease in the unit cost of treating patients. The extent to which these changes occurred as a result of TA remains unclear. Also, it remains unclear how costs will change longer-term.

The USD59 per year reduction in the unit cost of treatment between Phase I and Phase II does not appear to be heavily influenced by the introduction of TA. Several other factors appeared to be significant, including reductions in the price of ARVs and viral load tests, neither of which could be significantly associated with the introduction of TA (although the ability to negotiate for lower costs of ARVs and viral load tests might have been associated with increased need for these services given the introduction of TA and differentiated care models). However, it is unclear how to interpret the large decrease in personnel costs. The FTEs focused on ART located at each facility declined by 27% (on average) between Phase I and II, which was only partially offset by a 9% increase in government salaries. The reductions in personnel costs might optimistically suggest that as Namibia’s treatment program matures, it is becoming more efficient. It was observed that patient visits decreased, which might reflect healthier patients accessing HIV treatment services. Alternatively, it could suggest that the attention received by each patient is declining, perhaps suggesting a decline in quality. In either case, additional analysis is required to better understand what is driving reductions in personnel costs.

Second, the increase in the number of adult patients on ART at the sampled facilities amounted to only 3.8%. This wasn’t particularly surprising given that some facilities anticipated the change in policy and began treating all patients before the policy was officially adopted in April 2017. Thus, Phase I of the study did not report a significant number of pre-ART patients who were unable to access ARVs due to the existing policy. As a result, there apparently wasn’t a large “pent-up” supply of healthy patients who were waiting for the change in policy in April 2017. Patient numbers are also affected by the ongoing process of decentralization which results in the transfer of ART patients to lower-level facilities. The increase in total adult patients in all facilities in Namibia may therefore have been more significant with the initiation of TA, which was not reflected in the findings at these ten large and medium sized facilities.

### Potential implications of TA rollout in Namibia

The introduction of TA in most countries represents both an opportunity and a concern. The opportunity comes from treating patients more effectively by achieving viral suppression at an early stage of illness. The clinical evidence in favor of TA appears overwhelming. The concern arises from the potentially large investment of resources required to test and treat large numbers of additional people. This study should allay such fears at least in countries such as Namibia, as TA resulted in neither a large influx of patients nor an increase in unit costs. In fact, by focusing on the costs of ARVs and viral load tests, Namibia was able to reduce the unit cost of treatment.

#### Limitations and consequences of this approach

The methodology and the realities associated with hospital administrative systems’ weaknesses resulted in study limitations which may impact on results. First, the sample of ten facilities is small and is not representative of all health facilities and their ART activities in Namibia. Nor does the sample necessarily reflect the structure of the health system and the distribution of ART patients across the entire health system.

Next, the calculation of actual consumption of ARVs at site level was not possible given relatively poor stock records at the facilities. Actual consumption of ARVs may therefore be different to the quantities calculated based on the mix of patient regimens in each sampled facility. Any loss of ARVs (leakage or expiration of drugs) is not reflected in the study results.

Third, the inability to access detailed NIP records at facilities prevented the reporting of actual laboratory tests carried out. The actual cost of laboratory tests for ARV patients may have differed from the number of tests calculated based on the sample data.

Although the number of adult ART patients makes up more than 90% of total ART patients at the sampled facilities, it is interesting to note that the number of pediatric ART patients declined between Phase I and Phase II at these ten facilities (from 2,150 in Phase I to 798 in Phase II). This change may be due in part to pediatric patients being moved from the facilities in this study to other facilities that might be better equipped to treat pediatric ART patients. However, the reasons for this decline are not analyzed in this paper.

It is also worth noting that this analysis has focused on costs to the health system and not to the patients themselves. The treatment costs incurred by patients, including their travel time, opportunity costs, wait time, etc. are all important factors to be considered in evaluating the impact of TA. This study did not quantify these costs or evaluate how they changed with the introduction of TA.

Finally, several pharmaceutical products are used to combat OIs in ART patients. Most are not, however, used *exclusively* for ART patients. Even where stock records were available and reflected the total consumption of the list of prioritized OI pharmaceuticals, facilities were not able to split the commodities consumed between those consumed by ART patients and those consumed by non-ART patients. The cost of OI drugs included in this study are therefore an estimate provided by facility staff rather than an accurate reflection of the true consumption of OI drugs.

## Supporting information

S1 Data (XLSX)

S2 Data (ZIP)
